# Cellulose Nanofibers Improve the Performance of Retrograded Starch/Pectin Microparticles for Colon-Specific Delivery of 5-ASA

**DOI:** 10.3390/pharmaceutics13091515

**Published:** 2021-09-19

**Authors:** Andréia Bagliotti Meneguin, Rafael Miguel Sábio, Maurício Palmeira Chaves de Souza, Richard Perosa Fernandes, Anselmo Gomes de Oliveira, Marlus Chorilli

**Affiliations:** Department of Drugs and Medicines, School of Pharmaceutical Sciences, São Paulo State University (UNESP), Araraquara 14800-903, Brazil; andreia.meneguin@unesp.br (A.B.M.); rafael.m.sabio@unesp.br (R.M.S.); mauricio.palmeira27@gmail.com (M.P.C.d.S.); richard.p.fernades@unesp.br (R.P.F.); anselmo.gomes@unesp.br (A.G.d.O.)

**Keywords:** microparticles, spray dryer, colon-specific delivery, retrograded starch, 5-ASA, cellulose nanofibers

## Abstract

Cellulose nanofibers (CNF) were employed as the nanoreinforcement of a retrograded starch/pectin (RS/P) excipient to optimize its colon-specific properties. Although starch retrogradation ranged from 32 to 73%, CNF addition discretely disfavored the RS yield. This result agrees with the finding that in situ CNF reduces the presence of the RS crystallinity pattern. A thermal analysis revealed that the contribution of pectin improves the thermal stability of the RS/CNF mixture. Through a complete factorial design, it was possible to optimize the spray-drying conditions to obtain powders with high yield (57%) and low moisture content (1.2%). The powders observed by Field Emission Gum Scanning Electron Microscopy (FEG-SEM) had 1–10 µm and a circular shape. The developed methodology allowed us to obtain 5-aminosalicilic acid-loaded microparticles with high encapsulation efficiency (16–98%) and drug loading (1.97–26.63%). The presence of CNF in RS/P samples was responsible for decreasing the burst effect of release in simulated gastric and duodenal media, allowing the greatest mass of drug to be targeted to the colon. Considering that spray-drying is a scalable process, widely used by the pharmaceutical industry, the results obtained indicate the potential of these microparticles as raw material for obtaining other dosage forms to deliver 5-ASA to the distal parts of gastrointestinal tract, affected by inflammatory bowel disease.

## 1. Introduction

5-Aminosalicylic acid (5-ASA) is a non-steroidal anti-inflammatory drug belonging to the aminosalicylates class. It has been considered the drug of choice in the treatment of mild to moderate inflammatory bowel disease (IBD) [1], a set of autoimmune diseases characterized by chronic intestinal inflammations with causes not fully clarified [2] and whose main entities are ulcerative colitis and Crohn’s disease.

Considering the fast and extensive absorption of 5-ASA in the upper regions of the gastrointestinal tract (GIT) after oral administration, only an extremely low concentration of drug reaches the colon, reducing the local therapeutic effect [3]. In addition, the incidence of side effects after the rapid absorption of the drug in the upper portions of the GIT, such as diarrhea, nausea, abdominal pain, headache, vomiting and rash, frequently limit the patient’s adherence to treatment.

Some commercially available oral medications have been developed to overcome these limitations, such as the prodrugs sulfasalazine, olsalazine and balsalazine. Also available are delayed-release dosage forms, such as Eudragit S-coated 5-ASA tablets (Asacol^®^), which dissolve at pH ≥ 7, releasing the drug in the terminal ileum, and ethylcellulose-coated-5-ASA microespheres (Pentasa^®^) with a release mechanism dependent on the gastrointestinal transit time [4]. However, such medications may have reduced efficacy due to the insufficient activation time in cases of diarrhea for prodrugs [5], as well as the intra- and inter-individual variability of gastrointestinal pH and transit time for those based on polymers with pH and time-dependent solubility, respectively [6].

It is also known that the progress of IBD promotes important changes in the gastrointestinal physiology, with a marked decrease in pH values, which becomes moderately acidic for the affected colon (pH 2.3–5.5 against 6.0 to 7.2 for the normal colon) [7]. Such information clearly shows that the available medications for carrying 5-ASA are prone to performance errors, requiring the development of formulations that enable the 5-ASA targeting to the colonic region [8], making the approach more effective for the treatment of IBD [9,10].

Several technological methods have been used for the building of colon-specific systems, including fluidized bed coating [11], particle production by spray drying [12] and electrospraying [13], as well as fiber preparation using electrospinning [14,15]. Such methods are often combined with the use of natural substances, such as shellac (a resin secreted by female lac bugs) [13], starch [16], gellan gum [17], chitosan [18], pectins [19], xanthan gum, guar gum [20], hyaluronic acid [21] and alginate [22]. Among them, a modified starch known as retrograded starch (RS) is noteworthy, since its main targeting mechanism for the colon consists of specific enzymatic degradability in the colonic region [16,17,19].

The production of RS occurs through a green process combining gelatinization (disruption of the granular structure) and retrogradation (slow recrystallization of the starch components through storage and cooling) [23,24], leading to the construction of a more entangled three-dimensional network. This only occurs thanks to the formation of hydrogen bonds and van der Waals forces in order to achieve a metastable form of lower free energy [25] with the formation of double helices through junction zones [26].

Pectin (P) is another polysaccharide suitable to develop colon-specific delivery systems, since it is also degraded by colonic enzymes. In a first study, our research group evaluated the influence of P on starch retrogradation, revealing that its use in equal proportion to that of starch can promote up to 96% in retrogradation [19]. Moreover, the P addition resulted in a material with appropriate film-forming properties, absent in RS dispersions only. Thus, to prove the performance of RS/P film-forming dispersion, gellan gum microparticles loaded with insulin were coated, demonstrating the effective ability to control the blood glucose in diabetic rats after oral administration, with a reduction of up to 51% in blood glucose levels [17]. Despite the favorable results from a therapeutic point-of-view, in vitro release studies of RS/P coated-microparticles revealed the premature release of insulin in simulated gastric fluid (SGF) of 18–32%, attributed to the high P solubility [25].

Considering that gastro-resistant systems are characterized by releasing no more than 10% of the drug in SGF within 2 h [27], the improvement of the RS/P properties using cellulose nanofibers (CNF) as nanoreinforcement appears to be a promising technological strategy. It is known that nanofibrilated forms of cellulose show a dimensional nanoscale (20–50 nm diameter and ~100 μm in length) with a high surface area and aspect ratio, in which small amounts of CNF significantly improve the mechanical, thermal and control release properties of the drug delivery systems [28]. Particularly, regarding the drugs targeting the colon, CNF presents a peculiar behavior after drying, characterized by the attraction between the fibers through the establishment of hydrogen bonds among the hydroxylic groups on the cellulose surface, leading to the collapse of structures. Consequently, the CNF rehydration in the gastrointestinal fluids becomes difficult, contributing significantly to the prolongation of the release rates [29].

In a recent work, our research group used CNF to prepare microparticles via a spray-drying process using sodium diclofenac and caffeine as model drugs [30]. The samples showed a pH-dependent swelling and release behavior with reduced release rates in an acid medium (pH 1.2). Moreover, the sample developed with lower concentration of CNF (25%) showed significant gastroresistance (~13% of DS released after 120 min of acid-phase testing). Since there are no reports in the literature on the association of CNF with RS/P, it is our understanding that the employment of these two materials for the building of nanocomposite microparticles via spray drying can be considered an effective tool to optimize the performance of RS in the drugs targeting the colon.

## 2. Materials and Methods

### 2.1. Materials

High amylose starch (HAS) (type Hylon VII, 70% amylose and 30% amylopectin) was a gift from National Starch & Chemical (Bridgewater Township, NJ, USA), cellulose nanofibers (CNF) (HD Grade 2000, diameter ≤100 nm, 3.8% *w*/*w* dry content from Eucaliptus bleached kraft pulp) were supplied by Suzano Cellulose & Papel (Limeira, Brazil) and pectin (type LM-5206CS—DE < 50%) was provided by CP Kelco (Copenhagen, Denmark). Sodium hydroxide was supplied by Grupo Química (Rio de Janeiro, Brazil), and 37% hydrochloric acid, glucose and potassium phosphate monobasic (98.0–100.5%) were provided by Quimis (Diadema, Brazil). Pancreatin, polysorbate 80 and absolute ethanol were purchased from Vetec (Duque de Caxias, Brazil), and 3,5-dinitrosalicylic acid (DNS) (purity ≥ 98.0%) and 5-aminosalicylic acid (95%) were provided by Sigma-Aldrich Co. (St. Louis, MO, USA).

### 2.2. Starch Retrogradation

Starch was retrograded according to the methodology proposed by Meneguin et al. [19] with minor modifications. HAS was dispersed in distilled water at a concentration of 5% (*m*/*v*) under magnetic stirring for 30 min. Then, the HAS dispersion was autoclaved for gelatinization at 121 °C for 120 min. Next, the gelatinized HAS was cooled down to 30 °C and mixed with pectin (5%, *m*/*v*) and CNF (3.8%, *m*/*v*) in the following proportions: 1:1:0, 1:1:0.25, 1:1:0.50, 1:1:0.75 and 1:1:1 HAS:P:CNF, or 1:0.25, 1:0.5, 1:0,75 and 1:1 HAS:CNF. For the retrogradation process, the dispersions were submitted to alternating thermal cycles of 4 °C and 30 °C for 16 days, 2 days at each temperature.

### 2.3. Evaluation of CNF Influence on the Retrogradation Process

#### 2.3.1. Quantification of RS Content after Retrogradation

The in vitro enzymatic digestion of the samples prepared in Section 2.2 and the respective RS content were determined according to the method described by Englyst et al. [31]. Briefly, for every 100 mg of sample, 2 mL of phosphate buffer (0.1 M, pH 7.1) were added, kept at 100 °C for 30 min. Then, the samples were cooled to 37 °C, mixed with 0.5 mL of an enzymatic solution of pancreatin (0.15 g mL^−1^) and incubated at 37 °C [32]. During the incubation, aliquots (0.1 mL) were removed at predetermined intervals (20, 60, 120, 150 and 180 min) and mixed with 1 mL of 80% *v*/*v* ethanol to stop the enzymatic activity. A standard glucose curve was used, since starch is hydrolyzed to glucose, and its content quantified spectrophotometrically (λ = 540 nm) through a reaction with DNS. Starch digested within the first 20 min was termed as rapidly digestible (RDS), and starch digested between 20 and 120 min as slowly digestible (SDS). The RS content was calculated according to Equation (1) [19]:

(1) $$RS=\frac{\left(Starch_{total}-SDS-RDS\right)}{Starch_{total}}\times 100$$

#### 2.3.2. X-ray Diffraction Pattern (XRD)

Retrogradation process occurrence can be followed by narrowing peaks as well as the appearance of new crystallinity peaks observed in the XRD patterns. The identification of the crystalline and/or amorphous structure of the isolated polymers and retrograded samples was performed using diffractograms obtained in a Siemens^®^ X-ray diffractometer, model D5000 (Munique, Germany), with a goniometer speed of 0.05° s^−1^, under radiation Cu-Kα (λ = 1.5406 Å) and with 2θ open-angle X-ray scanning between 3° and 90°.

#### 2.3.3. Simultaneous Thermogravimetry and Differential Scanning Calorimetry (TG-DSC)

TG-DSC curves were obtained with a simultaneous thermal analyzer Mettler Toledo TG/DSC 1 (Columbus, OH, USA). A nitrogen atmosphere was used as purge gas with a 50 mL min^−1^ flow rate. A heating rate of 10 °C min^−1^ was adopted. The instrument was calibrated for temperature and heat flow using In and Zn. The samples weighed about 10 mg for each analysis, and alumina crucibles were used.

#### 2.3.4. Differential Scanning Calorimetry (DSC)

The enthalpy of events observed in TG-DSC until the thermodegradation process or 300 °C was measured with DSC Q10 (TA Instruments, New Castle, DE, USA). Nitrogen was used with a 50 mL min^−1^ flow rate and a heating rate of 10 °C min^−1^. Aluminum crucibles with perforated cover were used, with samples weighing approximately 2 mg.

### 2.4. Preparation of RS/P Microparticles by Spray Drying

Spray-dried microparticles were prepared in a mini spray-dryer model B-191 (Büchi, Flawil, Switzerland) in a concurrent flow regime, equipped with a peristaltic pump, a pneumatic spray nozzle and a cyclone for solids separation. Dispersions of RS/P (5% *m*/*v*; 1:1 ratio) were diluted to 2.5% (*m*/*v*), kept under magnetic stirring for 30 min before atomization and during the spray-drying process at 1000 rpm to keep them homogenized. The drying conditions, such as air inlet temperature, atomization air rate, aspiration and feed rate, were optimized to obtain a powder with adequate micromeritic and flow properties according to the factorial design presented below.

#### 2.4.1. Set-Up of Factorial Design

Aiming to find the best conditions of spray drying, the analysis was performed through a complete factorial design in which the inlet air temperature and aspirator factor was varied in three levels, followed by pump flow in two levels, forming the experiment matrix shown in Table 1, totaling 18 experiments (3^1^ × 3^1^ × 2^1^).

The critical quality parameters, residual moisture and process yield were examined. The optimization objective was to obtain the highest process yield with the lowest possible residual moisture, as well as to understand how the synthesis parameters can impact the characteristics of the systems obtained through the spray-drying technique. It is possible to establish a range and working conditions more suitable for the formulation of this material, as well as to establish a working space, which aims to reduce costs with the maintenance of product quality.

#### 2.4.2. Process Yield

Microparticles obtained after spray drying were immediately weighed, and the yield calculated in relation to the initial mass of solids. The results were performed in triplicate.

#### 2.4.3. Powder Moisture

The powder moisture content was measured by employing the halogen lamp analyzer (MB 45, Ohaus Co., Pine Brook, NJ, USA) at 105 °C for 30 min, and using 1.0 g of sample. The measurements were performed in triplicate.

#### 2.4.4. Powder Packing

Apparent bulk density (*ρ_bulk_*) was determined by pouring 2 g of dry powder into a 10 mL graduated cylinder and measuring the respective volume (*v_initial_*) (Equation (2)). Apparent tapped density (*ρ_tapped_*) was determined by submitting the cylinder to tapping until a constant volume (*v_final_*) was reached (Equation (3)). The analysis was determined according to the methodologies suggested by the United States Pharmacopeia [27]. All these determinations were performed in triplicate.

(2) $$\rho_{bulk}=\frac{m\;\left(g\right)}{v_{initial}\;\left(mL\right)}$$

(3) $$\rho_{tapped}=\frac{m\left(g\right)}{v_{final\;\left(mL\right)}}$$

#### 2.4.5. Powder Flowability

Hausner ratio (*H_r_*) and Carr’s index (*C_i_*) were used to estimate the flowability of spray-dried powders and were calculated using the measured values of *ρ_bulk_* and *ρ_tapped_* according to Equations (4) and (5) [27]. All these determinations were performed in triplicate.

(4) $$H_{r}=\frac{\rho_{tapped}}{\rho_{bulk}}$$

(5) $$C_{i}\left(\%\right)=\frac{\rho_{tapped}-\rho_{bulk}}{\rho_{tapped}}\times 100$$

#### 2.4.6. Field Emission Gun-Scanning Electron Microscopy (FEG-SEM)

FEG-SEM was performed using samples previously coated with evaporated carbon. The images were obtained using the JEOL JSM-7000 F microscope (Akishima, Tokyo, Japan) operating at 2 kV with 2000× magnification.

### 2.5. Performance Studies

#### 2.5.1. Preparation of 5-ASA-Loaded RS/P Microparticles

Dispersions of RS/P (1:1, 2.5% *m*/*v*) were mixed with the 5-ASA drug under different conditions. As 5-ASA is a drug with poor aqueous solubility and presents a pH-dependent solubility profile based on its three pKa values (2.15, 7.10 and 12.30), a study was first carried out only with the RS/P dispersion and the drug (without CNF addition), using different solubilization tools prior to the spray-drying process. The tests included (i) direct incorporation of 5-ASA (0.5% and 1.0%, *m*/*v*) in the RS/P dispersion (labeled as RS/P/5ASA0.5% and RS/P/5ASA1.0%, respectively), (ii) incorporation of 0.5% of 5-ASA in RS/P dispersion with pH < 2.0 (corrected with HCl and labeled as RS/P/5ASA-HCl), (iii) incorporation of 0.5% of 5-ASA in RS/P dispersion with pH 7.0 (corrected with NaOH and labeled as RS/P/5ASA-NaOH), and (iv) incorporation of 5-ASA in RS/P dispersion containing 2.0% (*v*/*v*) of polysorbate 80 (labeled as RS/P/5ASA-polysorbate). The sets were kept under magnetic stirring for 30 min before atomization and during the spray-drying process at 1000 rpm to keep them homogenized. The spray-drying conditions were selected from the factorial design (Experiment 1, Section 2.4.1) with 210 °C of inlet air temperature, 70% of aspirator and 0.05% of feed pump flow.

#### 2.5.2. Encapsulation Efficiency (EE%) and Drug Loading (DL)

The determination of EE% and DL were performed by dispersing 30 mg of the drug-loaded microparticles in 3 mL of phosphate buffer (0.1 M; pH 7.4) based on drug solubility. The set was maintained under magnetic stirring for 48 h and then centrifuged at 5000 rpm for 10 min. The supernatant was filtered on 0.45 μm nylon membranes prior to UV–vis quantification at 331 nm (Hewlett Packard 8452 Diode Array Spectrophotometer with Kayak XA workstation). For the quantification, calibration curves were constructed for 5-ASA in the linearity range between 5 and 50 μg mL^−1^, with a determination coefficient (*r*^2^) of 0.9999. The EE% and DL of each sample were evaluated in triplicate using Equations (6) and (7), respectively.

(6) $$EE\%=\frac{Quantified\;drug\;mass}{Theorical\;drug\;mass}\times 100$$

(7) $$DL=\frac{Mass_{5-ASA\;\left(mg\right)}}{100\;mg_{microparticles}}$$

#### 2.5.3. FEG/SEM

Possible morphological changes were verified in RS/P/5ASA samples, as well as the presence of unencapsulated drug crystals, using SEM/FEG according to Section 2.4.6, with 500× and 2000× magnifications.

### 2.6. Preparation of 5-ASA-Loaded RS/P/CNF Microparticles

After the selection of the better condition of 5-ASA incorporation based on yield, EE%, DL and morphology, dispersions of RS/P (1:1, 2.5% *m*/*v*) were mixed with different concentrations of CNF (10%, 25% or 50%, m/m in relation to the mass of RS/P) and 5-ASA (fixed at 0.5% *m*/*v* and directly added to the RS/P dispersion). The spray-drying conditions were the same as in Section 2.5.1. The samples were labeled as RS/P/5ASA-CNF10, RS/P/5ASA-CNF25 and RS/P/5ASA-CNF50 according to the CNF concentration (10%, 25% and 50%, respectively).

#### 2.6.1. In Vitro Drug Release

The in vitro 5-ASA release profiles from RS/P/CNF10, RS/P/CNF25 and RS/P/CNF50 microparticles were performed on a Hanson Research dissolution station (New Hanson SR-8Plus) equipped with a modified USP type I dissolution apparatus (mini-basket coated with dialysis bag (Mw cut- off 14,000 Da, Sigma-Aldrich^®^, St. Louis, MO, USA) and supplied with 150 mL of a small volume vessel, respecting the sink conditions. A precisely weighed mass of sample containing 10 mg of 5-ASA was used. In order to mimic the main pathophysiological conditions present in IBD, characterized by the acceleration of transit time and the reduction of pH values, the dissolution was performed according to the following three stages: the first one in simulated gastric fluid (SGF, 0.1 N HCl, pH 1.2) for 1 h, the second in simulated duodenal fluid (SDF, 0.1 M acetate buffer, pH 4.5) for 1 h, followed by the third step in simulated colonic fluid (SCF, 0.1 M phosphate buffer, pH 6.8) for 5 h. The set was stirred at 50 rpm and equilibrated at 37 °C. Aliquots of 2 mL were withdrawn in pre-determined intervals and replaced by a fresh medium at the same temperature. The samples were filtered through a 0.45 μm membrane prior to the quantitative analysis. The 5-ASA concentration was determined by UV-Vis spectroscopy from the calibration curves constructed for SGF, SDF and SCF, at 303, 302 or 331 nm, respectively. All experiments were performed in triplicate.

#### 2.6.2. Release Kinetics Models

From the release data obtained in Section 2.6.1, the mechanisms involved in the drug release process were analyzed through the application of several mathematical models (Baker-Londsdale, First-Order, Higuchi, Hixson-Crowell, Korsmeyer-Peppas and Weibull) [33,34,35] with the aid of Sigma Plot 10 software. Mathematical models were used throughout all the release profile, except for the Peppas and Weibull models, which used data referring to 60.0 and 63.2% of drug release, respectively.

## 3. Results and Discussion

### 3.1. Evaluation of CNF Influence on the Retrogradation Process

#### 3.1.1. Quantification of RS

RS can be considered a valuable material for targeting drugs to the colon, as it escapes digestion in the upper portions of the GIT. Since gelatinization causes the starch granules to break down, making them more amorphous, gelatinized starch is easily degraded by digestive enzymes. On the contrary, the RS digestibility is hampered because of the packing of amylose double helices, reducing the access of α-amylase to glycosidic bonds [36].

Different methodologies for RS quantification have been described. Generally, they vary as to the enzyme type, sample preparation and experimental conditions to mimic the GIT [37]. Methodologies such as those used in this work, that include steps of homogenization and heating of starch in the presence of buffer at 100 °C, have the advantage of eliminating RS-1 and RS-2 fractions from the total final RS value. The enzyme used to mimic digestion was pancreatin, a complex enzyme composed of amylase, lipase and protease, commonly used for this purpose [38].

**Figure 1:**
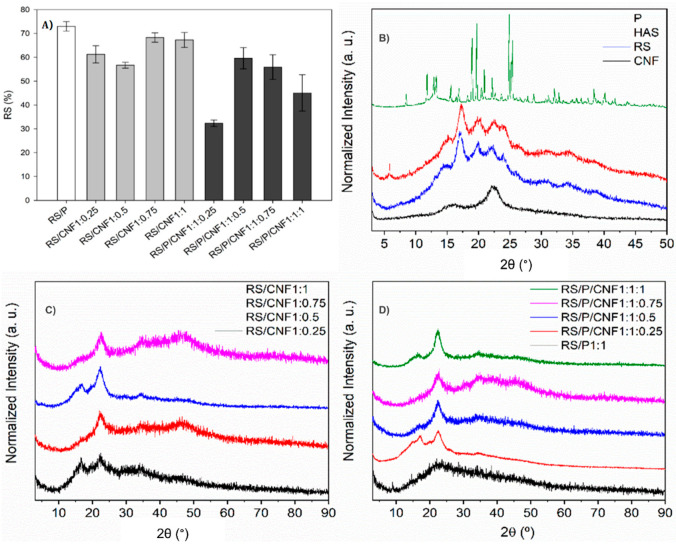
RS content (%) as a function of the different proportions of pectin and/or CNF after incubation with an enzymatic solution of pancreatin (0.15 g mL^−1^). RS quantification was performed through the DNS method for reducing sugar (**A**). X-ray diffractogram patterns of isolated polymers (P, HAS, RS and CNF) (**B**), RS in distinct proportions of CNF (RS/CNF1:1, RS/CNF1:0.75, RS/CNF1:0.5 and RS/CNF1:0.25) (**C**) and RS in the presence of P and distinct proportions of CNF (RS/P/CNF1:1:1, RS/P/CNF1:1:0.75, RS/P/CNF1:1:0.5, RS/P/CNF1:1:0.25) (**D**).

The conventional synthesis of RS/P (1:1 ratio) dispersions carried out according to Meneguin et al. (2014) provides already known RS contents, which ranged from 70 to 75% (Figure 1A). Considering the use of CNF as a strategy to increase the effectiveness of RS colon-specific systems, the knowledge of its influence during the retrogradation process is of great importance.

Figure 1A shows the data obtained after an in vitro enzymatic digestion test of retrograded samples. The sample with the highest RS content was RS/P (73%), and the addition of CNF both in the presence and in the absence of pectin disfavored the RS yield (ranged from 32.35% to 68.3%). Considering that the presence of several OH groups on the CNF surface makes it super reactive, supramolecular interactions with starch are expected, hindering the diffusion process of its molecules to build crystals [39]. This behavior was more evident in the samples containing both pectin and CNF (RS/P/CNF), which presented the lowest RS values, probably due to an increase in viscosity due to the presence of pectin, decreasing the crystal growth and nucleation.

Formulations containing higher RS levels are considered more effective in resisting to enzymatic digestion and targeting drugs to the colon. Thus, it was considered important to carry out retrogradation only in the presence of pectin (conventional method according to Meneguin et al. [19]), with the addition of CNF being done only immediately before spray drying.

#### 3.1.2. XRD

In starch gels, XRD studies supplemented with data from other techniques, clearly show increased crystallinity during storage [40]. The diffraction patterns of the samples are shown in Figure 1B–D.

The XRD pattern of CNF (Figure 1B) displayed characteristic peaks of cellulose crystalline phase at 16.2° and 22.5° (2θ), assigned to diffraction planes (101) and (002), respectively [16,41]. The XRD pattern of P (Figure 1B) exhibited intense and well-defined peaks at 12.7°, 16.72°, 18.42°, 25.32° e 40.14° (2θ) ascribed to its high crystallinity [19]. The HAS XRD pattern (Figure 1B) displayed peaks at 15°, 17.1°, 19.9°, 22.5° and 24° (2θ), characteristic of a B-type crystalline structure, with the highest intensity found at 17.1° (2θ), suggesting a highly ordered crystal structure of lipid-amylose complexes in starch granules [42]. Although the XRD pattern of RS seemed similar to the HAS, a discrete peak at 13° (2θ) can be assigned to the V-type crystalline structure achieved after the retrogradation process of HAS [19].

According to the diffractograms of the RS/CNF samples (Figure 1C), all samples exhibited XRD patterns with broad peaks at 16.7°, 22.5°, 34.4° and 46.5° (2θ), suggesting the presence of RS and CNF patterns. It is worth emphasizing that the peak intensity at 22.5° (2θ) increases as a function of the increase of CNF concentration. Similar profiles were detected for RS/P/CNF samples (Figure 1D) with broad peaks at 16.9°, 22.5°, 34.6° and 46.1° (2θ), characteristic of the overlapping of RS, CNF and P diffraction patterns [16,19]. In addition, it was noted that increasing the CNF content, the peak intensity at 22.5° (2θ) increases regarding the other peaks. These results are in agreement with the RS content results, showing that the increase of CNF concentrations in situ can reduce the presence of the RS XRD pattern and then the retrogradation process.

#### 3.1.3. TG/DSC

**Figure 2:**
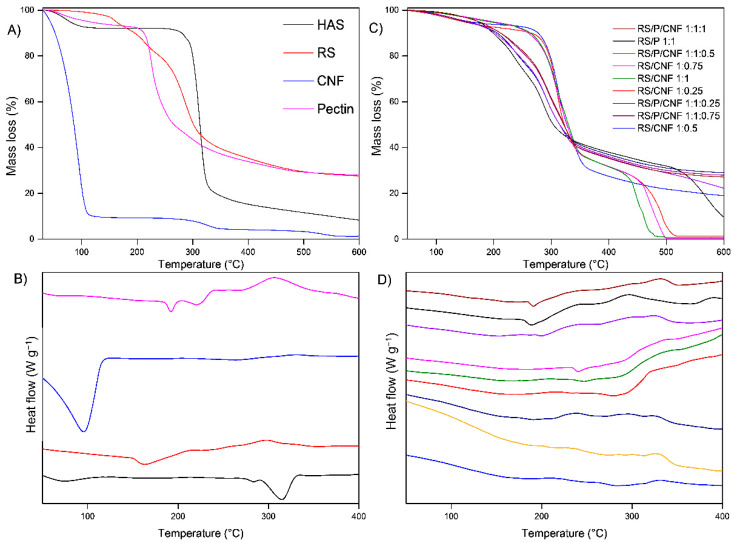

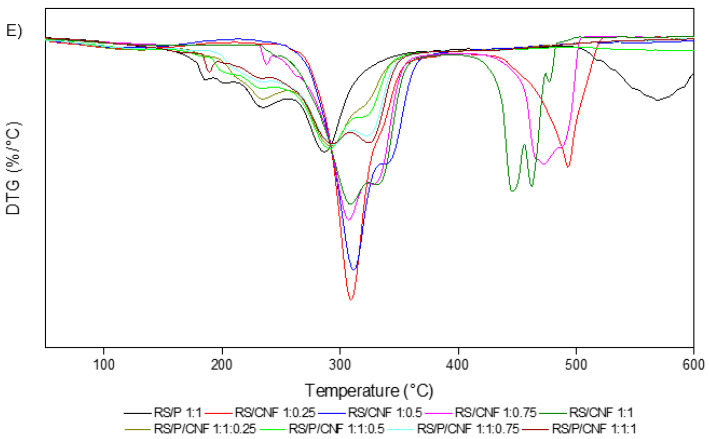
TG-DSC curves of isolated compounds and samples retrograded in the presence of pectin and/or CNF. TG curve (**A**) and DSC curve (**B**) of RS, HAS, Pectin, CNF. TG curve (**C**) and DSC curve (**D**) of RS/CNF and RS/P/CNF in different proportions. DTG curve of the RS in presence of different P and CNF proportions (**E**).

Isolated compounds exhibit a profile similar to that of previously reported data. The TG-DSC curves of RS, HAS, Pectin, CNF and retrograded samples (with or without pectin and CNF) are shown in Figure 2A–D.

The TG-DSC curves of HAS indicate a dehydration between 30 and 130 °C (Δm = 7.80%), which is related to the moisture content evaporation attributed to the endothermic peak at 73 °C in the DSC curve. Above this temperature, the sample becomes stable up to 230 °C, and the thermal decomposition occurs between 230 and 335 °C (Δm = 71.6%) related to two endothermic peaks at 283 and 315 °C, attributed to depolymerization followed by amylose starch decomposition [43].

The TG curve of RS shows a mass loss between 30 and 150 °C (Δm = 2.9%) related to moisture, followed by an endothermic peak in the DSC curve at 162 °C attributed to the loss of chemically bonded water between 150 and 195 °C (Δm = 6.96%). Thermal degradation occurs above this temperature with events relative to depolymerization [43,44].

The pectin TG-DSC curve shows an endothermic peak at 68 °C, attributed to dehydration with mass loss of 5.3% in the TG curve between 30 and 120 °C. Above this temperature, the thermal decomposition occurs between 175 and 400 °C (Δm = 59.3%) and 400 and 600 °C (Δm = 5.8%), related to the endothermic peaks at 191 °C and 220 °C, followed by some exothermic events attributed to the depolymerization of pectin chains at higher temperatures [45].

The CNF TG-DSC curves show that the sample exhibits water evaporation between 30 and 127 °C (Δm = 90.3%) related to the sharp endothermic peak at 93 °C in DSC curve. The second and third steps of mass loss between 221 and 400 °C (Δm = 5.5%) and 400 and 600 °C (Δm = 3.0%) are related to the decomposition of hemicellulose and cellulose [46].

The TG-DSC curves of the mixtures exhibit a different behavior, based on the components used. Looking for the differences observed for each system, DTG curves of the mixtures were plotted and are shown in Figure 2E. According to DTG, the RS/CNF mixtures exhibit three decomposition steps, well defined between 30 and 212 °C, 212 and 386 °C and 386 and 500 °C. In terms of mass loss, RS/CNF 1:0.25 exhibits the higher mass loss, between 240 and 370 °C, followed by RS/CNF 1:0.5, RS/CNF 1:0.75 and RS/CNF 1:1. In addition, moisture evaporation occurs predominantly in RS/CNF 1:0.25 and RS/P/CNF 1:1:0.25, while RS/P 1:1 exhibits the lowest values of mass loss, between 30 and 150 °C.

### 3.2. Factorial Design

Powder yield is an important indication of the drying parameters’ adequacy. However, the process recovery in lab-scale spray dryers was shown to be dependent on several factors, such as liquid properties [47], feed rate, air volume and process temperature. The recovery of the dried product is strongly affected by the dryer chamber, the cyclone engineering and the product’s properties and processing conditions [48,49]. Besides, the stickiness of the droplets to equipment wall depends on their hygroscopicity, glass transition temperature, moisture and thermal diffusivity [50].

In this regard, the results of the process yield (%) and moisture (%) of the RS/P spray-dried samples were chosen to determine the better conditions of spray drying before adding the drug and CNF. We considered the variables inlet air temperature, aspirator efficiency and feed flow rate, presented in Table 1, as experiments from 1 to 18. The values found for the process yield are acceptable for a lab-scale spray-drying process [51]. The main concept of a lab-scale spray dryer is to design the chamber as small as possible, which considerably reduces the yield or powder recovery [48,49,51].

In the literature, a yield of 50% has been deemed as very reasonable [51], and most published papers report even lower yields [52]. The minimized chamber size and wall deposition resulting from the stickiness of some materials are mainly responsible for the low yields observed during spray-drying processes. Nevertheless, this problem should be of no concern, since higher yields can be expected in larger or industrial scales, and it can also be overcome by adding drying additives with a high glass transition point [47,48,51,52].

**Figure 3:**
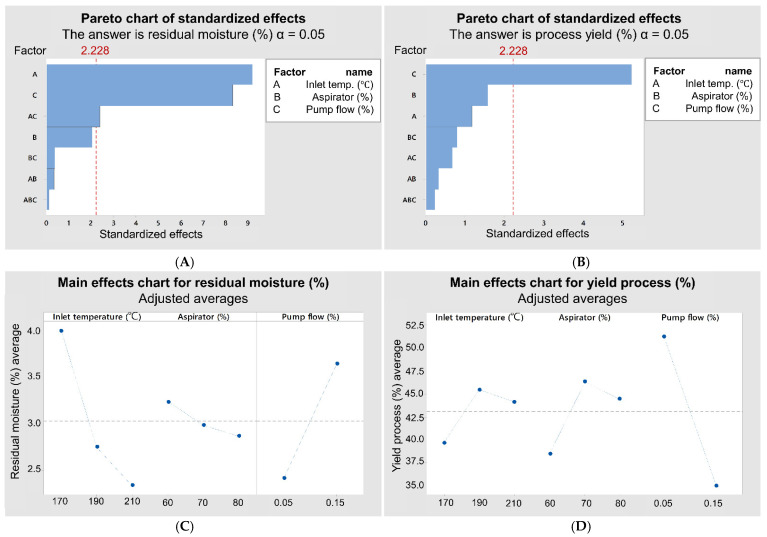
Pareto chart representing the impact of critical synthesis parameters under residual moisture (**A**) and process yield (**B**) and graphic representation of individual factors and their impact on the residual moisture (**C**) and yield process (**D**) factors.

From the results of the factorial design, it was possible to establish a correlation between input and output variables, and the data can be seen in Figure 3A–D. In Figure 3A,B, it is possible to identify which of the input parameters had the greatest impact on the residual moisture and process yield, respectively.

The analysis of Figure 3A,B shows that for both output parameters, different factors significantly impact the process. In Figure 3A, it is possible to identify that inlet temperature and pump flow have a more significant impact on the residual moisture of the systems. Additionally, it is also possible to verify the effect of the interaction between these two factors on the system moisture. Regarding the process yield (Figure 3B), only the pump flow variable significantly impacts this parameter. The simultaneous evaluation of the graphs indicates that pump flow is important for the two response variables.

As described above, the aspirator efficiency does not impact the critical quality parameters evaluated in the systems. This behavior can be reinforced in Figure 3C, in which there was no great variation in the averages when this factor was changed. However, variations of 170–210 °C for inlet temperature generated systems with residual moisture ranging from 2.5 to 4.0%, and the higher the temperature, the lower the residual moisture (Figure 3C). On the other hand, pump flow showed a fluctuation on the residual moisture of 2.5–3.7% when it varies from 0.05 to 0.15% (Figure 3C).

The inlet temperature exerts a major influence on the residual moisture because the drying operation is a thermodynamic process based on the transfer of temperature to the material, causing the solvent evaporation in the sample [53]. Thus, the inlet temperature has a direct effect on the heat and mass transfer during the drop-drying process. Comparatively, higher temperatures can promote greater solvent evaporation, a fact that was evidenced in the present study. However, excessively high temperatures associated with high temperature transfer rates can promote the rapid evaporation of the moisture, forming a porous and dry layer with high thermal resistance. As a result, the heat transfer to the innermost regions of the particles is impaired, originating irregular surfaces and moister cores [54,55].

Tonon et al. [56] have already demonstrated that higher feed rates result in a higher moisture content in the final particles. Another observation commonly used for spray-dryer studies is that low feed rates generate higher yields, a fact that can be confirmed in Figure 3D.

Regarding the effects of inlet temperature and aspirator parameters, it is not possible to verify any clear trend on the process yield, which can be explained by the fact that these variables had no significant effect on the process. On the contrary, decreasing the feed pump flow from 0.15% to 0.05% improved the process yield by 57.1%. This behavior occurred because with a reduction in the pump flow, a smaller amount of sample fed the equipment, providing an adequate heat transfer rate. A suitable condition of heat transfer has been considered of extreme importance, as it prevents the material from sticking to the equipment wall before it has completely dried [57].

Considering that the required quality patterns are a high process yield with the low residual moisture, the parameters that best fit are those from experiment 1 (Table 1), that is, 210 °C of inlet air temperature, 0.05% feed pump flow and 70% aspirator.

### 3.3. Powder Packing and Flowability

Table 2 shows the *ρ_bulk_*, *ρ_tapped_*, *H_r_* and *C_i_* for samples spray-dried at different inlet temperatures, aspirator capacities and feed flow rates. Comparing the values obtained with the reference values of the flowability scale [27], it is concluded that regardless of the employed variable, all samples present the impaired flow demonstrated trough the low bulk and tapped densities, and high *H_r_* (from 1.81 to 2.50) and *C_i_* (45–60%). This behavior is generally related with the reduced size and high surface area of the particles engineered by spray drying, which becomes more cohesive because of strong interparticular interactions. The presence of irregularly shaped particles also contributed to the challenges in the handling and processing. However, it is known that a hydrophobic surface could improve the flowability, and the addition of drying additives such as magnesium stearate can overcome this limitation [58].

### 3.4. FEG/SEM

**Figure 4:**
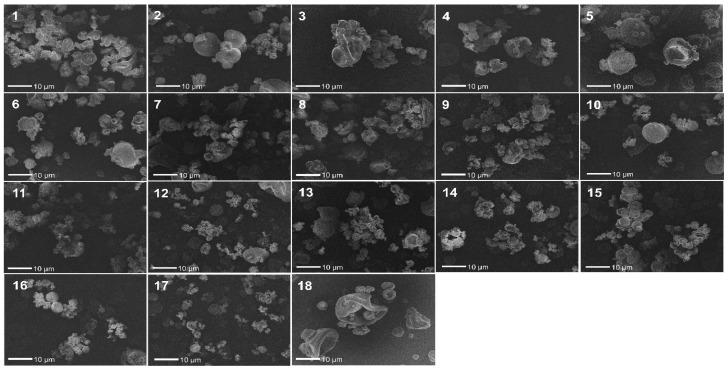
FEG-SEM images of RS/P samples prepared by spray drying employing different values for inlet air temperature, aspirator efficiency and feed flow rate, according to the factorial design outlined in Table 2. The images were obtained with 2000× magnification.

Figure 4 shows the FEG-SEM images of RS/P samples with size varying between 1 and 10 µm. All samples, regardless of the treatment used (experiments from 1 to 18), showed an approximately circular shape, characteristic of the samples obtained by spray drying. In this process, the feed polymer solution is atomized into droplets with a high surface area which quickly turn into dry particles, respecting the droplets’ shape [59].

### 3.5. Spray-Drying Process of 5-ASA-Loaded RS/P

#### 3.5.1. Encapsulation Efficiency (EE%) and Drug Loading (DL)

The 5-ASA microencapsulation by spray drying can be considered effective, since it originated spherical microparticles with high EE% and DL. As can be seen in Table 3, all samples showed an EE% greater than 90%, regardless of the type of treatment applied. Interestingly, the only exception was the sample containing the surfactant polysorbate 80 (RS/P/5ASA-polysorbate), which reduced the EE% to 47%. Despite the recognized activity of this compound in improving the water-solubility of many drugs, this sample had a very sticky aspect in addition to its low process yield, which indicates that there were losses due to its adherence to the equipment walls, also impacting on EE%.

#### 3.5.2. FEG/SEM

**Figure 5:**
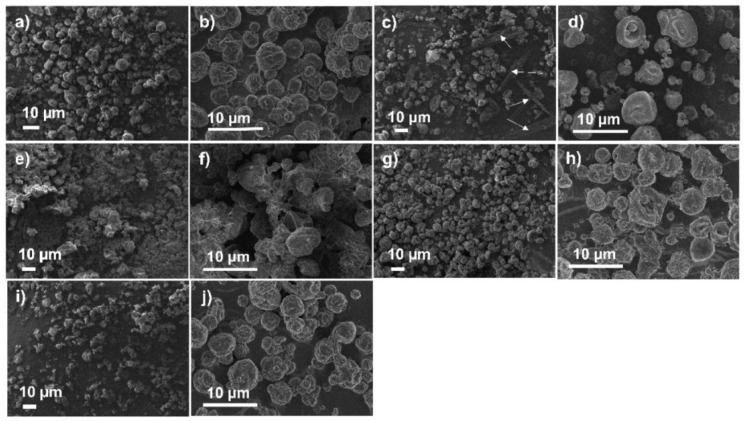
FEG-SEM images of 5-ASA-loaded RS/P samples prepared by spray drying employing different strategies to improve drug solubility before the drying process: RS/P5ASA0.5% with 500× (**a**) and 2000× magnification (**b**), RS/P5ASA1.0% with 500× (**c**) and 2000× magnification (**d**), RS/P5ASA-HCl with 500× (**e**) and 2000× magnification (**f**), RS/P5ASA-NaOH with 500× (**g**) and 2000× magnification (**h**), and RS/P5ASA-polysorbate with 500× (**i**) and 2000× magnification (**j**). White arrows indicate the presence of crystals as free drug (unencapsulated).

The images obtained by SEM (Figure 5) clearly show that the addition of 5-ASA was responsible for obtaining more homogeneous particles in terms of size (monodisperse) and with a greater degree of circularity, suggesting the success of the microencapsulation process. Such behavior was expected, since drug molecules at ideal concentrations (such as 0.5 mg mL^−1^) fill the empty spaces between polymer chains, promoting the construction of denser and more organized structures. On the other hand, it was possible to observe that particles developed with the highest concentration of 5-ASA (1 mg mL^−1^, Figure 5c,d) were deformed, in addition to showing drug crystals in unencapsulated form, indicating the inability of the system to encapsulate an excess of drug.

Considering that the RS/P/5ASA0.5% sample presented a high sphericity, the absence of free drug crystals, and an EE% higher than 90%, the preparation process of RS/P/5ASA0.5% (without pH correction or surfactant addition) was selected for further experiments. Such choice should also contribute to the minimization of possible interferences in the results related with the presence of the additives.

### 3.6. Spray-Drying Process of 5-ASA-Loaded RS/P/CNF

After selecting the better conditions of spray drying (experiment 1 of the factorial design, Section 3.2) and 5-ASA incorporation (Section 3.5), the final microparticles containing CNF were developed and tested regarding their drug delivery performance. RS/P/5ASA-CNF10 and RS/P/5ASA-CNF25 showed high values of EE% (about 95%) and DL (13.2–14.7 _mg5-ASA_/100 mg microparticles, respectively) (Table 3), while a significant decrease in these parameters was observed for the RS/P/5ASA-CNF50. It is known that increased concentrations of nanofibrillated forms of cellulose significantly increase the viscosity of polymer dispersions, making the technological process of spray drying difficult in reason of the greater water affinity of CNF and the nozzle clogging.

**Figure 6:**
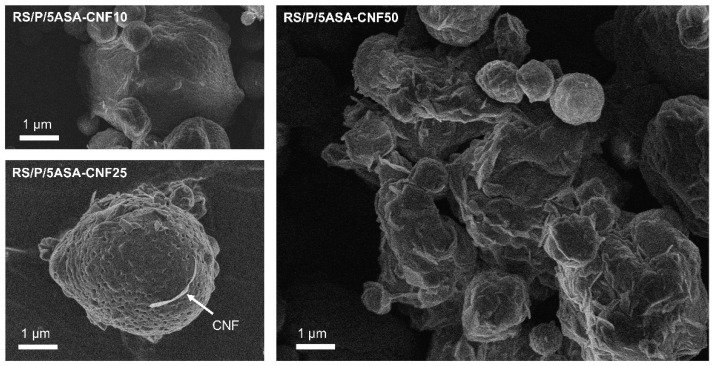
FEG/SEM images of 5-ASA-loaded RS/P samples containing different amounts of CNF (10%, 15% and 50%). The images were obtained with 10,000× magnification.

The presence of CNF on the microparticles’ surface appeared proportionally to the increase in its concentration, with the less concentrated sample (RS/P/5ASA-CNF10) having a smooth surface, while the sample containing a higher concentration of CNF (RS/P/5ASA/CNF-50) was rougher due to the surface coating with the collapsed nanofibers (Figure 6). In the latter case, microparticles were more flatted in shape than their counterparts, probably because it followed the same trend of nanofibers morphology, that is, larger in extension than in diameter [30]. It is also believed that a synergistic effect occurred between the increased viscosity of RS/P/5ASA/CNF-50 (which generates bigger droplets and different drying kinetics) and the rigidity of the collapsed nanofibers on particles’ surface, flattening them.

#### 3.6.1. In Vitro 5-ASA Release

**Figure 7:**
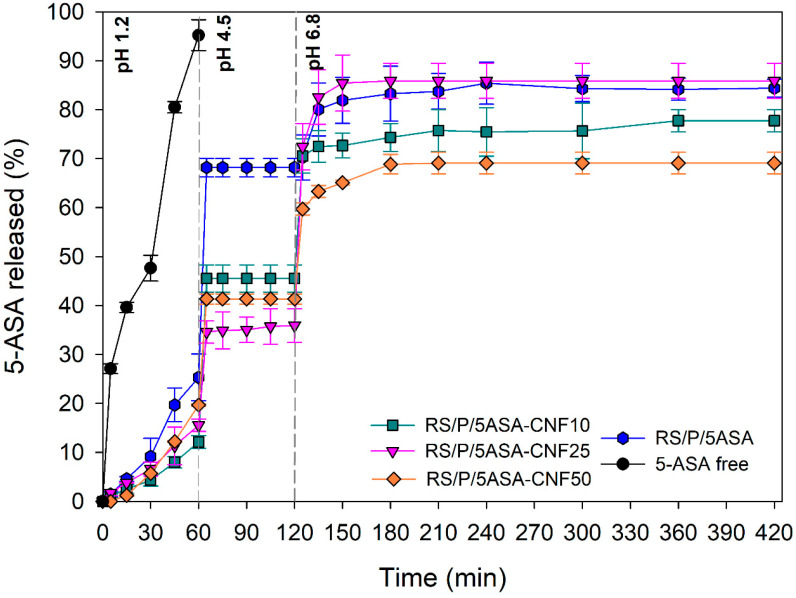
In vitro 5-ASA release profiles from spray-dried microparticles in media with different pH values, mimicking gastric (0–60 min; pH 1.2), duodenal (60–120 min; pH 4.5) and colonic (120–480 min; pH 6.8) phases. Each value represents the mean ± standard deviation, n = 3.

An in vitro 5-ASA release test from microparticles was performed employing a pH gradient to mimic the pH variations imposed along the particles’ passage through the GIT. The total dissolution of free 5-ASA occurred within 60 min in a gastric medium, which is in accordance with the high drug solubility in media with pH < 2 (Figure 7).

As noted earlier, the RS/P blend is an important candidate for drug delivery to the colon. Its performance has already been analyzed from free films [19], RS/P tablets [60], RS/P-coated microparticles [17], RS/gellan microparticles [61] and nano-in-RS/P microparticles [62]. Interestingly, the spray-dried sample RS/P/5ASA (original formulation, not optimized with CNF) did not show resistance to pH changes, releasing approximately 25% of 5-ASA in SGM, followed by 68% in SDM, demonstrating its inability to deliver drugs to the colon. It is believed that this unexpected behavior is a result of polymers/drug amorphization induced by the spray-drying technique, facilitating the dissolution processes. The small size of the particles obtained by spray drying (<10 µm) should also contribute to such behavior, since particles with a high surface area interact more easily with the dissolution media. However, it was important to confirm the initially suggested hypothesis that CNF could act by optimizing the RS/P blend. Different release profiles could be obtained through the different concentrations of CNF added to the system, and in all of them the highest drug load was released when encountering the SCM.

After 60 min of test in acid medium, we observed the release of approximately 12%, 15% and 25% of 5-ASA for samples RS/P/5ASA-CNF10, RS/P/5ASA-CNF-25 and RS/P/5ASA-CNF-50, respectively, indicating that the higher the CNF addition, the higher the drug release. However, a distinct behavior was observed in a duodenal medium in which the intermediate CNF concentration was more effective in controlling the 5-ASA release rates from RS/P/5ASA-CNF25 (only 34% of release after 120 min).

Interestingly, all samples had a release burst effect when the medium was changed to SDM and SCM, but were followed by a plateau during the entire time of analysis at these pH values. Such behavior highlights not only the pH-dependent release of RS/P/CNF, but also the release triggered trough the digestion ability of enzymes from colonic microbiota. Thus, the developed samples can be considered effective, since they allow 55 to 65% of the drug load added to the system to be fully targeted to the colon.

#### 3.6.2. Release Kinetic

From the determination coefficient values (*r*^2^) presented in Table 4, the mathematical model that best correlated with the release data of the studied systems was the Weibull model. Weibull’s mathematical model exponentially relates the fraction of drug released, Mt/M∞, as a function of time t (COSTA; LOBO, 2001), and according to the value of the b parameter, the mechanism involved in drug release can be classified as Fickian diffusion (*b* ≤ 0.75), non-Fickian diffusion associated with Case-II (0.75 < *b* < 1) and a complex release mechanism (*b* > 1) [17].

For the RS/P/5ASA sample, both the Korsmeyer–Peppas and the Weibull models presented high *r*^2^ values (0.9887 and 0.9793, respectively). The first model is based on the power law and exponentially relates drug release with time. The n value found, 1.28, is indicative of a Super-Case II transport, highlighting the influence of polymer hydration and the relaxation of polymer chains on drug release. In agreement with this finding, the value of *b* > 1 found through the Weibull model describes drug release as a complex mechanism involving diffusion, swelling and erosion.

The samples containing CNF correlated better with the Weibull model with both RS/P/5ASA-CNF10 and RS/P/5ASA-CNF50 showing 0.75 < *b* < 1, indicative of non-Fickian diffusion (case-II transport), in which the dominant mechanism for drug transport is due to polymer matrix relaxation. However, it was interesting to note that the intermediate concentration of CNF (25%) in the RS/P/5ASA-CNF25 sample changed the release mechanism for Fickian diffusion (*b* < 0.75).

Such a drastic change in the 5-ASA release mechanism suggests that specific concentrations of CNF lead to the construction of different structuring patterns when associated with polymers. In the case of RS/P/5ASA-CNF25, it was possible to observe the presence of many pores on the surface of microparticles (Figure 6) which favor the diffusion according to a concentration gradient through water-filled pores [63], without the contribution of swelling. Kolakovic et al. [64] reported early the ability of nanofibrilated cellulose to form porous microparticles through the spray-drying process.

## 4. Conclusions

In order to optimize the performance of an RS/P-based colonic excipient, CNF was added as a nanofiller for further building nanocomposite microparticles via spray drying. The starch retrogradation process in the presence of CNF was impaired, most likely due to the establishment of supramolecular interactions between these two compounds that hinder the conformational freedom of starch chains for recrystallization in a more resistant form. However, the presence of CNF in the spray-dried microparticles was essential to reduce the burst of 5-ASA release in simulated gastric and duodenal media, favoring the colon-specific release of 5-ASA. These results set precedents for further research using the RS/P excipient optimized with CNF as a multiparticulate raw material to obtain new dosage forms, such as tablets and capsules. Such medications would represent a great advance in relation to market-available drugs, aiming at a localized and more effective treatment of inflammatory bowel disease.

## Figures and Tables

**Table 1:** Design matrix of the factorial experiment and results of the yield process and moisture content of the spray-dried samples.

Experiments	Inlet Air Temperature (°C)	Aspirator (%)	Pump Flow (%)	Process Yield (%)	Residual Moisture (%)
1	210	70	0.05	57.1 ± 0.8	1.2 ± 0.2
2	210	80	0.05	52.5 ± 1.1	1.3 ± 0.2
3	210	60	0.05	48.9 ± 0.7	1.8 ± 0.1
4	190	70	0.05	55.1 ± 0.5	2.2 ± 0.1
5	190	80	0.05	54.4 ± 0.5	2.1 ± 0.3
6	190	60	0.05	40.0 ± 0.9	2.5 ± 0.5
7	170	70	0.05	57.6 ± 0.4	3.6 ± 0.1
8	170	80	0.05	52.0 ± 0.6	3.3 ± 0.4
9	170	60	0.05	43.2 ± 1.3	3.7 ± 0.2
10	210	70	0.15	40.8 ± 1.0	3.2 ± 0.3
11	210	80	0.15	36.0 ± 0.8	3.1 ± 0.1
12	210	60	0.15	29.6 ± 0.3	3.4 ± 0.5
13	190	70	0.15	38.4 ± 1.4	3.2 ± 0.3
14	190	80	0.15	40.0 ± 0.7	3.0 ± 0.2
15	190	60	0.15	44.8 ± 0.9	3.5 ± 0.7
16	170	70	0.15	28.9 ± 1.0	4.5 ± 0.6
17	170	80	0.15	31.8 ± 0.5	4.4 ± 0.3
18	170	60	0.15	24.5 ± 1.5	4.5 ± 0.4

**Table 2:** Results of the apparent bulk density (*ρ_bulk_*), apparent tapped density (*ρ_tapped_*), Hausner ratio (*H_r_*) and compressibility index (*C_i_*) of spray-dried samples under different conditions according to the factorial design.

Sample	*ρ_bulk_* (g mL^−1^)	*ρ_tapped_* (g mL^−1^)	*H_r_*	*C_i_* (%)
1	0.202 ± 0.001	0.504 ± 0.005	2.50 ± 0.03	60 ± 1.5
2	0.267 ± 0.001	0.593 ± 0.003	2.22 ± 0.05	55 ± 0.9
3	0.236 ± 0.004	0.535 ± 0.001	2.27 ± 0.01	56 ± 0.4
4	0.261 ± 0.002	0.544 ± 0.001	2.08 ± 0.12	52 ± 1.0
5	0.147 ± 0.006	0.293 ± 0.001	2.00 ± 0.06	50 ± 1.0
6	0.126 ± 0.006	0.279 ± 0.007	2.22 ± 0.03	55 ± 1.6
7	0.128 ± 0.001	0.285 ± 0.005	2.22 ± 0.05	55 ± 0.3
8	0.180 ± 0.009	0.328 ± 0.010	1.81 ± 0.01	45 ± 0.6
9	0.156 ± 0.008	0.301 ± 0.008	1.92 ± 0.01	48 ± 1.1
10	0.132 ± 0.003	0.274 ± 0.002	2.08 ± 0.07	52 ± 0.9
11	0.145 ± 0.004	0.291 ± 0.002	2.00 ± 0.02	50 ± 0.8
12	0.146 ± 0.004	0.292 ± 0.013	2.00 ± 0.02	50 ± 0.2
13	0.136 ± 0.012	0.273 ± 0.004	2.00 ± 0.11	50 ± 0.3
14	0.284 ± 0.002	0.558 ± 0.005	1.96 ± 0.08	49 ± 1.2
15	0.230 ± 0.006	0.524 ± 0.001	2.27 ± 0.01	56 ± 1.1
16	0.293 ± 0.007	0.606 ± 0.006	2.06 ± 0.05	51 ± 1.4
17	0.299 ± 0.002	0.612 ± 0.003	2.04 ± 0.04	51 ± 0.9
18	0.261 ± 0.005	0.575 ± 0.004	2.19 ± 0.10	54 ± 0.5

**Table 3:** EE% and DL results of 5-ASA from spray-dried RS/P using different strategies to solubilize the drug before spray drying and from samples containing different amounts of CNF. The results were expressed as average ± standard deviation (n = 3).

Sample	EE%	DL (*mass*_*5-ASA* (*mg*)_/100 *mg_microparticles_*)
RS/P/5ASA0.5%	93.72 ± 8.2	15.61 ± 1.36
RS/P/5ASA1.0%	93.23 ± 6.21	26.63 ± 1.77
RS/P/5ASA-HCl	98.73 ± 9.97	16.45 ± 1.66
RS/P/5ASA-NaOH	90.71 ± 2.48	15.11 ± 0.41
RS/P/5ASA-polysorbate	47.19 ± 1.23	7.86 ± 0.20
RS/P/5ASA-CNF10	95.81 ± 2.29	14.75 ± 0.35
RS/P/5ASA-CNF25	95.93 ± 3.37	13.28 ± 0.46
RS/P/5ASA-CNF50	16.76 ± 0.85	1.97 ± 0.10

**Table 4:** Release coefficients of mathematical release models (Baker–Lonsdale, Higuchi, Korsmeyer–Peppas, First order, Hixson–Crowell and Weibull) from spray-dried microparticles.

Release Models			Samples		
		RS/P/5ASA	RS/P/5ASA-CNF10	RS/P/5ASA-CNF25	RS/P/5ASA-CNF50
Baker–Lonsdale	K (% min^−1^)	0.0009	0.0005	0.0006	0.0004
	*r* ^2^	0.8119	0.7933	0.7519	0.8309
Higuchi	K (% min^−1/2^)	5.4173	4.5807	4.8873	4.1571
	*r* ^2^	0.7526	0.7982	0.7824	0.8270
Korsmeyer–Peppas	K (% min^−n^)	0.1317	0.2660	0.4667	0.2807
	*r* ^2^	0.9887	0.7945	0.8649	0.9033
	*n*	1.2800	1.1112	0.9387	1.0960
First order	K (1 min^−1^)	0.0098	0.0063	0.0068	0.0053
	*r* ^2^	0.8824	0.8721	0.8635	0.8708
Hixson–Crowell	K (%^1/3^ min^−1^)	0.0028	0.0018	0.0020	0.0015
	*r* ^2^	0.8671	0.8471	0.8727	0.8185
	*r* ^2^	0.9793	0.9916	0.9716	0.9213
	*b*	1.3550	0.7636	0.0070	0.8246

## Data Availability

Data is contained within the article.
